# COVID-19 Disease and Vitamin D: A Mini-Review

**DOI:** 10.3389/fphar.2020.604579

**Published:** 2020-12-17

**Authors:** Mohamed Said Boulkrane, Victoria Ilina, Roman Melchakov, Julia Fedotova, Filippo Drago, Lucia Gozzo, Undurti Narasimha Das, A. M. Abd El-Aty, Denis Baranenko

**Affiliations:** ^1^ International Research Centre “Biotechnologies of the Third Millennium”, ITMO University, Saint-Petersburg, Russia; ^2^ Laboratory of Neuroendocrinology, I.P. Pavlov Institute of Physiology, Russian Academy of Sciences, St. Petersburg, Russia; ^3^ Lobachevsky State University of Nizhny Novgorod, Nizhny Novgorod, Russia; ^4^ Department of Biomedical and Biotechnological Sciences, Biological Tower, School of Medicine, University of Catania, Catania, Italy; ^5^ UND Life Sciences, Battle Ground, WA, United States; ^6^ Department of Pharmacology, Faculty of Veterinary Medicine, Cairo University, Giza, Egypt; ^7^ Department of Medical Pharmacology, Medical Faculty, Ataturk University, Erzurum, Turkey

**Keywords:** SARS-CoV-2, COVID 19, respiratory tract infection, vitamin D3, vitamin D3 receptor

## Abstract

Novel coronavirus disease (COVID-19) pandemic caused by SARS-CoV-2, for which there is no effective treatment except employing prevention strategies, has already instituted significant number of deaths. In this review, we provide a scientific view on the potential role of vitamin D in SARS-CoV-2 virus/COVID-19 disease. Vitamin D is well-known to play a significant role in maintaining the immune health of an individual. Moreover, it induces antimicrobial peptide expression that can decrease viral replication and regulate the levels of pro-inflammatory/anti-inflammatory cytokines. Therefore, supplementation of vitamin D has the potential to reduce the incidence, severity and the risk of death from pneumonia resulting from the cytokine storm of many viral infections including COVID-19. We suggest that supplementation of subjects at high risk of COVID-19 with vitamin D (1.000 to 3.000 IU) to maintain its optimum serum concentrations may be of significant benefit for both in the prevention and treatment of the COVID-19.

## Introduction

The occurrence of respiratory tract infections (RTI) is more common in winter, especially in the northern regions, than in the summer months (Hope-Simpson, 1981). This also applies to the rapidly spreading in the winter period around the world of the infectious Coronavirus disease 2019 (COVID-19) which became a pandemic, since the virus is more easily transmitted at low temperatures (Qu et al., 2020; Sajadi et al., 2020). This rises the possibility that insufficient intake of vitamin D_3_ may have a role in the development and severity of COVID-19. Thus, in order to curb the current pandemic of COVID-19, it is opined that the administration of an adequate amounts of vitamin D_3_ may stem the current situation till an effective therapy, chemoprophylaxis, and vaccination is developed.

Deficiency of vitamin D_3_ in all age groups is a public health problem (Palacios and Gonzalez, 2014) that is well recognized. It is estimated that more than one billion people suffer from vitamin D_3_ deficiency (Van Schoor and Lips, 2011). Several previous studies suggested that there is an independent association between low plasma concentrations of 25-hydroxyvitamin D_3_ and susceptibility to acute respiratory infections (Cannell et al., 2006). Vitamin D_3_ deficiency has been associated with many diseases including but not limited to type 2 diabetes mellitus, heart disease, stroke, autoimmune diseases, asthma and RTIs (Hollick, 2007; Hollick, 2017). The relation between low levels of vitamin D_3_ and infection with bovine diarrhea virus in calves has been well established (Nonnecke et al., 2014). It is evident that in winter due to the shorter time spent in the sun, the plasma levels of vitamin D_3_ is likely to be low (Berardi and Newton, 2009; https://www.medlineplus.gov/vitamind.html). This is especially evident in countries such as the United States of America (USA), United Kingdom (UK), Switzerland, Italy, Spain, Iran, France, Turkey, etc. It is rather interesting that COVID-19 pandemic and its high mortality (Pharmacy Times, 2020; https://www.pharmacytimes.com/publications/issue/2010/february2010/otcfocusvitamind-0210) has been reported in these countries. According to the US National Center for Health Statistics, approximately 70% of the population may be deficient in vitamin D_3_ and surprisingly while the United States is presently the most affected by COVID-19 (Kmiec et al., 2014). This is in line with the current proposal that severe acute respiratory syndrome due to SARS-CoV-2 and its associated high mortality rate may be as a result of vitamin D_3_ deficiency. Furthermore, vitamin D_3_ deficiency is known to elevate with increasing age and comorbidities that are associated with lower vitamin D_3_ levels.

In the current review, we present a scientific rationale on the potential relationship between vitamin D_3_ content and higher incidence of Severe Acute Respiratory Syndrome Coronavirus-2 (SARS-CoV-2) virus infection. Moreover, our review also summarizes the current understanding of the link among vitamin D_3_, the immune system, and respiratory infections.

## Vitamin D and Immune System

Vitamin D is a pluripotent hormone that modulates the innate and adaptive immune responses (Rezaei, 2018). Vitamin D could play a decisive role in the proliferation and immunomodulation of cells, affecting several immune pathways enhancing the protective properties of the mucous membranes of the body and inhibiting excessive inflammation (D’Ambrosio et al., 1998; Khare et al., 2013; Parlak et al., 2015). Immunocytes such as macrophages, B and T lymphocytes, neutrophils and dendritic cells express Vitamin D_3_ receptors (VDRs) that is enable to the actions of vitamin D (Di Rosa et al., 2011). The active metabolite of vitamin D lead to the activation of VDRs that can form Retinoid X Receptor (RXR) heterodimer that, in turn, influences the proteins of the innate and adaptive immune system (the regulatory T cells, defensins, cytokines, pattern recognition receptors, etc.) (Chun et al., 2014).

The immune system is influenced in various ways by both vitamin D_3_ and its metabolite 1,25-hydroxy-vitamin D_3_. 1,25-hydroxy-vitamin D_3_ rigorously regulates antimicrobial peptides such as defensin and cathelicidin (Adams et al., 2009). Cathelicidin possesses an antimicrobial function against mycobacteria, Gram-positive and Gram-negative bacteria due to its ability to destroy cell membranes. 1,25-hydroxy-vitamin D_3_ has antiviral effect against adenovirus, herpes simplex virus, enveloped and non-enveloped retroviruses, and fungi (Herr et al., 2007). By damaging cell membranes, these peptides penetrate infected cells and neutralize the action of endotoxins (Agier et al., 2015). For instance, the LL-37, antimicrobe peptide, has antibacterial and antifungal properties by virtue of its ability to disrupt the integrity of the cell membrane and proton gradient (Bals and Wilson, 2003) by vitamin D_3_ (Howell et al., 2004; Leikina et al., 2005; Steinstraesser et al., 2005; Bergman et al., 2007). In addition, vitamin D_3_ inhibits the production of pro-inflammatory cytokines and augments that of anti-inflammatory cytokines (Gombart et al., 2020). Thus, vitamin D_3_ influences the incidence and severity of viral infections by altering the production of pro-inflammatory cytokines. There is reasonable evidence to suggest that vitamin D_3_ can inhibit the transcription induced by tumor-necrosis-factor-α (TNF-α) in latently infected cells by human immunodeficiency viruses (HIV) (Nunnari et al., 2016). These and other results suggest that vitamin D_3_ can inhibit the production of inflammatory cytokines and chemokines such as TNF-α, interferon-β (IFN-β), interleukine (IL)-8, IL-6 and Regulated upon Activation, Normal T Cell Expressed and Presumably Secreted (RANTES) (Hansdottir et al., 2010; Khare et al., 2013). Increase in mortality in those with COVID-19 is due to acute respiratory distress syndrome (ARDS) due to unantagonized production of pro-inflammatory cytokines IL-6 and TNF-α. Vitamin D_3_ has a decisive role in the regulation of the innate and adaptive immune responses implying that adequate intake of vitamin D_3_ may protect patients with COVID-19 at least, in part by inhibiting the excess production of IL-6 and TNF-α (Daneshkhah et al., 2020). Vitamin D_3_ can also contribute to the modification of the antiviral response by enhancing the secretion of pro-inflammatory chemokines (C-X-C Motif Chemokine Ligand 8, CXCL8 and C-X-C Motif Chemokine Ligand 10, CXCL10) (Brockman-Schneider et al., 2014). Lytic phase of cytomegalovirus (CMV) replication can be induced by vitamin D_3_ *in vitro* (Wu and Miller, 2015).

Vitamin D_3_ promotes immunoglobulin and complement-mediated phagocytosis by stimulating the maturation of monocytes to macrophages. In addition, vitamin D_3_ maintains self-tolerance by reducing a hyperactive adaptive immune system (Bowie and Unterholzner, 2008). Vitamin D_3_ reduces the replication of influenza A (Barlow et al., 2011), rotavirus (Zhao et al., 2019) and dengue microbes (Martínez-Moreno et al., 2019). These results imply that excess innate immune response induced by viral and other microbial infections seen in patients with SARS-CoV-2 and associated cytokine storm can be effectively reduced by vitamin D_3_ (Huang et al., 2020). The immunomodulatory effect of vitamin D_3_ on viral infections appears to be temporary and at least, this in part could be attributed to its immunomodulatory role in viral infections is rather complex and depends on the nature of the pathogen and the type of immune function that is needed to resolve the disease process (Sacco et al.,2012; Gotlieb et al., 2018).

There is reasonable evidence to suggest that vitamin D_3_ modulates adaptive immune responses by inhibiting the Th1 cell function that leads to a reduction in the production of TNF-α, IL-2, granulocyte macrophage colony-stimulating factor and IFN-β. 1,25-(OH)_2_-Vitamin D_3_ enhances the action of Th2 cells and production of their anti-inflammatory cytokines, IL-4, IL-5, and IL-10 (Hughes and Norton, 2009). In addition, supplementation of vitamin D_3_ increases the number of regulatory T cells (Treg cells), suppresses IgG production and differentiation of dendritic cells (Kamen and Tangpricha, 2010; Aranow, 2011; Rondanelli et al., 2018). 1,25-(OH)_2_-Vitamin D_3_ inhibits the proliferation and activation of T cells and T and B lymphocytes (Martineau et al., 2017). Thus, vitamin D_3_ suppresses T-cell-mediated inflammation and promote the proliferation of Treg cells that results in an increase in the production of IL-10 that leads to suppression of inappropriate inflammation (Adorini and Penna, 2009; Chun et al., 2014). Vitamin D_3_ can also increase the expression of glutathione reductase and glutamate–cysteine ligase modifier subunit (Lei et al., 2017) that may lead to a decrease in oxidative stress. These results led to the proposal that (Biancatelli et al., 2019; Mousavi et al., 2019; Wimalawansa, 2020) vitamin D_3_ may be of benefit to combat SARS-CoV-2 infection (Grant et al., 2020a).

Vitamin D deficiency is common in patients with HIV (Herr et al., 2007). The antiviral action of vitamin D_3_ can also be attributed to its ability to increase the production of cathelicidin and defensins (Herr et al., 2007; Hughes and Norton, 2009; Beard et al., 2011). Furthermore, 1,25-dihydroxy-cholecalciferol is known to regulate more than 200 genes including those responsible for cell proliferation, differentiation, and apoptosis (Umar and Sastry, 2018) including those involved in immune homeostasis (Van Herwegen et al., 2017). Recent meta-analysis of randomized controlled trials (RCTs) showed that vitamin D deficiency increases the overall mortality (Bjelakovic et al., 2014; Keum et al., 2019; Manson et al., 2019; Scragg, 2020). All above-mentioned effects of Vitamin D_3_ are presented in Table 1.

**TABLE 1:** Some effects of vitamin D on the immune system.

Immune cell type	Effect of vitamin D	References
Airway epithelium	Increases CD14 and cathelicidin. Dampens IFN-β and chemokine response during viral infection	Hansdottir et al. (2010)
Alveolar macrophages	Increases the antimicrobial peptide cathelicidin	Liu et al. (2007)
Dendritic cells	Inhibits dendritic cell differentiation, maturation and function, decreases IL-12 and increases IL-10, alters T cell activation	Penna and Adorini (2000); Piemonti et al. (2000); Fritsche et al. (2003); Sigmundsdottir et al. (2007)
T lymphocytes	Inhibits proliferation, modulates cytokine production - inhibits Th1 and Th17 cytokines but induces Tregs	Lemire et al. (1995); Penna and Adorini (2000); Sigmundsdottir et al. (2007); Daniel et al. (2008); Mora et al. (2008)
B lymphocytes	Inhibits proliferation of activated B cells and generation of plasma cells	Chen et al. (2007)

## Relevance of Vitamin D Regarding to Respiratory Tract Infections and Influenza

There is a provided evidence given by many reviewed studies to support the hypothesis that higher serum level of vitamin D_3_ is associated with a low risk of microbial infections and deaths from RTIs caused by pneumonia and influenza. In addition, SARS-CoV-2 infection and decrease the severity and mortality may be avoided by a normal serum vitamin D_3_ levels (Wimalawansa, 2020). Unfortunately, there are no standard recommendations regarding the dose and the desired optimal concentration of vitamin D_3_ required to protect people from RTI during the winter season.

Epidemiological studies revealed that vitamin D_3_ plays a critical role in viral RTIs and associated acute lung injury (Hansdottir and Monick, 2011). In a recent meta-analysis, it has been shown that a daily or weekly vitamin D_3_ dose between 20 and 50 μg resulted in a significant reduction of RTIs (Martineau et al., 2017). A high-dose, isolated, or added bolus of (2.5 mg once or monthly) did not reduce the risk. One study supplemented for one-year high risk individual for ARDS with a 100 μg/daily (Bergman et al., 2012). The overall infection score was significantly reduced in the treated groups, and those with vitamin D_3_ deficiency showed the greatest benefit of the supplementation.

In addition, it is observed that the degree of protection generally increases when the concentration of vitamin D_3_ reaches its optimal range of 40 to 60 ng/ml. To reach this level, an individual must take between 2,000 and 5,000 IU/day of vitamin D_3_ (Heaney et al., 2003). Calcitriol protects against acute lung injury by modulating the expression of the renin–angiotensin system including angiotensin-converting enzyme 2 (ACE2) in lung tissue (Xu et al., 2017). There seems to be a direct relationship between plasma 25-(OH)-Vitamin D_3_ concentrations and severity of COVID-19 (Huang et al., 2020; Wang et al., 2020; Zhou et al., 2020). It is noteworthy that the expression of the DPP-4/CD26 receptor is significantly reduced as a result of vitamin D_3_ deficiency (Komolmit et al., 2017). Furthermore, adequate provision of vitamin D_3_ seems to attenuate immunological events that may lead to prolonged interferon-gamma response (Zdrenghea et al., 2017), and persistent interleukin six elevation that are negative prognostic value indicators in those with severe COVID-19 (Miroliaee et al., 2018).

VDRs are very widely distributed in respiratory epithelial cells and immune cells (B cells, T cells, macrophages and monocytes). VDRs are in the epithelium of the bronchi and immune cells (Pfeffer and Hawrylowicz, 2012). The enzyme, 1a-hydroxylase (CYP27B1), required for vitamin D activation, is induced by diverse stimuli, including cytokines and toll-like receptor ligands in the respiratory tract. Nevertheless, adequate serum levels of 25-(OH)-vitamin D_3_ is required to increase levels of 1,25-(OH)_2_-vitamin D_3_ and to improve the immune response to respiratory virus infections (Greiller and Martineau, 2015). The development of ARDS shows typical changes in membrane permeability of the alveolar capillary, progressive edema, severe arterial hypoxemia and pulmonary hypertension (Matthay et al., 2012). In animal studies, vitamin D_3_ significantly attenuated lung damage caused by lipopolysaccharides (LPS) (Xu et al., 2017). This is noteworthy since LPS increase the pulmonary expression of renin and angiotensin 2 (Ang 2) that promotes inflammation. Vitamin D_3_ reduces the increased renin and Ang 2 expression and thus significantly lowers lung injury. It has been suggested that vitamin D_3_ promotes ACE2/Ang 1–7 activity. This is supported by the observation that calcitriol treatment significantly increased the expression of VDR mRNA and ACE2 mRNA that leads to a reduction in angiotensin II, ACE2 expression resulting in suppression of inflammation (Yang et al., 2016). VDRs are not only a negative regulator of renin, but also of NF-kB (Li et al., 2004), leading to an increase in Ang 2 formation, which promotes pro-inflammation (Jurewicz et al., 2007).

Down-regulation of ACE2 expression by SARS-CoV infection is associated with acute lung damage (edema, increased vascular permeability, reduced lung function) and associated RAS dysregulation leads to increased inflammation and vascular permeability as seen in COVID-19 (Imai et al., 2005). It was reported that COVID-19 is associated with release of pro-vitamin D_3_ enhances the cellular immunity and reduces the cytokine storm induced by the innate immune system. Vitamin D_3_ can reduce the production of pro-inflammatory cytokines such as TNF-α and IF-γ (Tjabringa et al., 2005; Baeke et al., 2010; Laaki, 2012). Several studies showed that adequate intake and plasma levels of vitamin D_3_ reduces the risk of viral infections through their action on immunocytes (Carnell et al., 2006; Baeke et al., 2010; Schwalfenberg, 2011; Lang and Samaras, 2012). Hence, it is suggested that vitamin D_3_ may have a significant role in COVID-19 due to its action on T cells (Zhang et al., 2015).

Type-II pneumocytes which are the primary target of coronaviruses, express high levels of ACE2 receptor (Bombardini and Picano, 2020). Metabolites of 25-(OH)-vitamin D_3_ have been reported to stimulate surfactant synthesis in alveolar type-II cells (Rehan et al., 2002). Human fetal and adult alveolar type-II cells supplemented with 1,25-dihydroxy-vitamin D_3_ show increased levels of VDRs and expression of surfactant associated protein B, a lipid-associated protein of the pulmonary surfactant, indicating the potential of vitamin D_3_ to reduce surface tension in COVID-19 (Phokela et al., 2005).Comorbid conditions such as diabetes mellitus, hypertension and chronic obstructive pulmonary disease are commonly associated with low plasma vitamin D_3_ levels (Malinovschi et al., 2014; Kim et al., 2015; Grant et al., 2020a). Hence, it is reasonable to propose that COVID-19 may be associated with low plasma vitamin D_3_ levels. Hence, it is suggested that vitamin D_3_ supplementation may be of significant benefit in COVID-19. Grant, in the latest report, suggest that vitamin D level checking will be conducted only in as elected category of patients that involves pregnant mothers, obese and elderly people and others suffering from certain comorbid conditions (Grant et al., 2020b). Multiple factors such as an ability of the assimilation by the gastrointestinal tract, body weight, genetic factors and the baseline 25-(OH)-vitamin D_3_ concentration, control the increase in vitamin D concentrations with respect to oral vitamin D_3_ supplementation. Given the degree of vitamin D_3_ deficiency, taking 5,000 IU of vitamin per day, it could be essential to elevate 25-(OH)-vitamin D_3_ levels to 40 ng/ml by (Veugelers et al., 2015).

A recent article indicates that vitamin D_3_ value >20 ng/ml is required and this advice is adopted by several countries (Amrein et al., 2020). Another research suggests a higher dose for RTIs, indicating rates >30 ng/ml of vitamin D_3_ as effective in decreasing cancer incidence, unfavorable pregnancy and birth outcomes and type 2 diabetes mellitus (Grant et al., 2020b). From another analysis it is suggested that optimal vitamin D_3_ standard should be 40–60 ng/ml for prevention of breast and colorectal cancer (Garland et al., 2009).

The U.S. Institute of Medicine noted that no research observed negative consequences of supplementation of vitamin D_3_ of less than 10,000 IU/daily, but set the upper consumption limit at 4,000 IU/daily, partially owing to retrospective tests that found U-shaped 25-(OH)-vitamin D_3_ concentration/health outcome relationships. However, further findings indicate that most observations of J- or U-shaps relationships came from observational studies that did not test serum 25-(OH)-vitamin D_3_ concentrations, and that the likely explanation for these relationships was the presence of some participants who started taking vitamin D_3_ complementation shortly before registration (Grant et al., 2016). Particularly in winter, supplementation with vitamin D_3_ is required for many individuals to reach concentrations of 25-(OH)-vitamin D_3_ above 30 ng/ml (Pludowski et al., 2018). However, vitamin D_3_ fortification of basic foods such as dairy and flour products may increase serum 25(OH)D concentrations by a few ng/ml among those members of different populations with the lowest concentrations (Pilz et al., 2018; Grant and Boucher 2019). This will contribute to a decreased risk of ARTIs for persons with a severe vitamin D_3_ deficiency (Camargo et al., 2012; Martineau et al., 2017). However, regular or weekly treatment of vitamin D_3_ is advised for greater benefits (Martineau et al., 2017), as is the annual evaluation of serum 25-(OH)-vitamin D_3_ levels for health risks individuals (Grant et al., 2020b).

Table 2 describes the findings from meta-analyses that vitamin D_3_ is protective against acute RTI, particularly in patients with vitamin D_3_ deficiency.

**TABLE 2:** The finding on the efficacy of vitamin D in the respiratory tract infections.

Participants	Study characteristics	Vitamin D effect	References
5,660 participants (age ranging from 6 months to 75 years)	Eleven randomized placebo-controlled trials	Supplementation with vitamin D significantly decreased the risk of RTI (OR: 0.64; 95% CI: 0.49, 0.84; *p* = 0.0014)	Bergman et al. (2013)
1,868 participants (aged 1–83 years)	Five clinical trials	The reduction of episodes of RTI was significantly lower in vitamin D supplementation group compared to the control group (OR = 0.58; 95% CI: 0.42, 0.81; *p* = 0.001)	Charan et al. (2012)
10,933 participants (aged 0–95 years) from 14 different countries	Twenty five randomized controlled trials	Overall results showed that vitamin D supplementation has protective effective in decreasing the risk of suffering at least one acute RTI (OR 0.88; 95% CI: 0.81, 0.96; *p* = 0.003)	Martineau et al. (2017)

OR, Odds ratio; RTI, Respiratory tract infection; CI, Confidence interval.

## Hypothesis of the Correlation on Vitamin D_3_ Levels and Coronavirus Disease-19 Cases/Severity

Still there is a lack of a cohort studies and clinical trials in determining the role of vitamin D_3_ in the prevention of COVID-19 infections and/or severity. Some retrospective studies have demonstrated the relationships between vitamin D_3_ levels and COVID-19 cases and severity (Table 3).

**TABLE 3:** The outcomes in recent studies about the correlation of vitamin D_3_ concentrations with COVID-19 infections.

Country	Population type	n	Study design	Vitamin D_3_ doses	Outcomes	Reference
Singapore (a tertiary academic hospital)	Adults, age ≥50 years	43	Cohort observational	Vitamin D_3_ 1,000 IU, Mg 150 mg, and vitamin B_12_ 500 μg (oral)	i) A fewer patients who received vitamin D_3_, Mg and vitamin B_12_ required the subsequent oxygen therapy compared to controls (3/17 vs. 16/26, p = 0.006)	Tan et al., 2020
					ii) in multivariate analysis, the patients treatment with vitamin D_3_, Mg and vitamin B_12_ have showed a significant protective effects against clinical deterioration (p = 0.041) after adjusting for age, gender and comorbidities	
20 European countries	Adults	Cases and death/1 M population	Retrospective	NA	A significant negative correlation was observed for the serum 25-(OH)-vitamin D_3_ levels with COVID-19 cases (p = 0.033) but not with a death (p = 0.123) per million of population	Present study
20 European countries	Adults	Cases and death/1 M population	Retrospective (as of 8 April 2020)	NA	A negative correlation was observed between the serum 25-(OH)-vitamin D_3_ levels and COVID-19 cases (p = 0.050) and a death (p = 0.053) per million of population	IIie et al. (2020)
Southern Asian countries	NA	222	Retrospective multicentral study	NA	i) The differences in the levels of vitamin D_3_ mean were significant within the mild, ordinary, severe and critical cases of COVID-19 (p < 0.001)	Alipio (2020)
					ii) Vitamin D_3_ status showed a significant association with clinical outcomes (p < 0.001)	
USA (a single tertiary academic medical center)	Adults, mean age 65.2 years	20	Retrospective observational study	NA	A high vitamin D_3_ insufficiency was observed in ICU patients (84.6%) than in the floor patients (57.1%) (p = 0.29)	Lau et al. (2020)
South Asia (two tertiary medical centers)	Adults, age ≥60 years	176	Retrospective	NA	i) Severe patients had a low level of vitamin D than mild patients	Glicio et al. (2020)
					ii) Subjects with the pre-existing medical conditions had a low level of vitamin D_3_	
UK (UK Biobank data 2006–2010 for vitamin D_3_ and ethnicity)	Adults, age 37–73 years	449	Cross-sectional (16 March–14 April 2020)	NA	i) Vitamin D_3_ levels showed a significant association with COVID-19 infection in an univariate analysis (p = 0.013) but not after an adjustment for confounders (p = 0.208)	Hastie et al. (2020)
					ii) Ethnicity showed a significant association with COVID-19 infection univariably	
United Kingdom (UK Biobank data 2006 2010 for BMI, vitamin D_3_ and ethnicity)	Adults, mean age 57.7 years	580 cases and 723 control	Retrospective	NA	i) No significant difference was observed for vitamin D_3_ levels between COVID-19 cases and the control group	Darling et al. (2020)
					ii) Vitamin D_3_ status was significantly lower in those of Asian, Black and mixed ethnicity (p < 0.0010) compared with those of White ethnicity	
					iii) Vitamin D_3_ levels were significantly lower in those with obesity (p < 0.001). Overweight or obese person; living in London; being male and being of Asian, Black or mixed ethnicity was associated with a higher odd of positive cases	
					iv) In the regression model, the interaction between BMI and vitamin D_3_ status did not predict the test result in the available data set	
Mainland of United States (48 states and Columbia district)	1.609.488 cases and 91.094 deaths	-	Retrospective (22 Jan–23 May 2020)	NA	i) Latitudes were marginally associated with the cases (p = 0.0792) and the deaths (p = 0.0599)	Li et al. (2020)
					ii) Sunlight and vitamin D_3_, with latitude as an indicator, possibly associated with reduced risks for both COVID-19 cases and mortality	
Belgium (central network hospital)	Adults, median age 71 years (cases), 68 years (control)	186 cases, 2,717 controls	Retrospective observational (1 March–7 April 2020)	NA	i) Patients with COVID-19 had significantly a low median value of vitamin D_3_ and higher vitamin D_3_ deficiency compared to control subjects (p = 0.0016, p = 0.0005, respectively)	De Smet et al. (2020)
					ii) This difference were more pronounced in male COVID-19 subjects than male control subjects that increased with advancing radiological stage and were not confounded vitamin D_3_-impacted comorbidities	
Hospitals and clinics from different parts of the world	Age up to 80 years	5,000 cases	As on March 21, 2020	NA	About 15% reduction in the number of severe COVID-19 cases given a normal vitamin D_3_ status within a population	Daneshkhah et al. (2020)
Indonesia (Government hospital)	Adults, mean age 54.5 years	780 cases	Retrospective cohort study (2 March 2–24 April 2020)	NA	i) In univariate analysis, older and male cases with the pre-existing medical condition and below normal vitamin D_3_ levels were associated with the higher odds of death	Raharusun et al. (2020)
					ii) After adjustment of confounders (age, sex and comorbidity), vitamin D_3_ levels showed a strong relationship with the COVID-19 mortality	

For example, a preliminary information study from Philippines on 212 reported COVID-19 patients, found that the severity of the infection is a highly correlated to the vitamin D_3_ levels (Alipio, 2020). Authors have found that 85.5% of patients with an adequate status of vitamin D_3_ (>30 ng/ml) showed a moderate disease, while a 72.8% of patients with vitamin D_3_ deficiency (<20 ng/ml) had the serious disease symptoms (Alipio, 2020). The correlation between vitamin D_3_ and COVID-19 have extensively investigated in a group of 178 Indonesians (Raharusun et al., 2020). According to this study, the patients with vitamin D_3_ levels in the categories, 20–30 and <20 ng/ml, were 12.55 times and 19.12 times more likely to die from COVID-19, respectively, as compared with COVID-19 patients with sufficient levels of vitamin D_3_. The main conclusion is that, even after controlling for age, sex and comorbidities, deaths were 10.12 times more likely in patients with vitamin D_3_ deficiency than in patients with normal vitamin D_3_ levels (Raharusun et al., 2020). A limited cohort observational study with 43 cases in Singapore have found that a treatment of COVID-19 patients with an oral doses of vitamin D_3_ (1.000 IU), Mg (150 mg), and vitamin B_12_ (500 μg) significantly reduced the application of the subsequent oxygen therapy compared to controls (3/17 vs. 16/26, *p* = 0.006) (Tan et al., 2020). Furthermore, such drugs combination have protected against the clinical deterioration (p = 0.041) even after adjustment of confounders (age, sex and comorbidity) (Tan et al., 2020). Severe COVID-19 patients and patients with pre-existing medical conditions were reported to have low levels of vitamin D_3_ (Glicio et al., 2020; Lau et al., 2020). A retrospective observational study with 186 positive cases and 2717 negative controls in Belgium have demonstrated a low median for vitamin D_3_ in the COVID-19 patients compared to the control subjects (*p* = 0.0016) (De Smet et al., 2020). A retrospective cohort study with 780 cases in Indonesia showed that below-normal vitamin D_3_ levels and the pre-existing medical conditions in the older and male cases have higher odds of death. Moreover, the vitamin D_3_ status has a strong relationship with COVID-19 mortality if it adjusted for age, sex and comorbidities (Raharusun et al., 2020). The similar retrospective study in the USA with many cases have showed that the reduced risks for both COVID-19 cases and the mortality are possibly associated with the sunlight and vitamin D_3_, as well with the latitude as an indicator (Li et al., 2020).

In a new systematic review and meta-analysis with an ecological approach, they found a high percentage of COVID-19 patients who suffer from vitamin D_3_ deficiency or insufficiency. Much more important its ecological investigation resulted in the substantial direct and reverse correlations between the recovery and mortality rates in COVID-19 patients with vitamin D_3_ deficiency at the different countries. A small reverse correlation between vitamin D_3_ status and the mortality rate have found globally. The populations with a lower levels of vitamin D_3_ might be more susceptible to the novel coronavirus infection (Ghasemian et al., 2020). Recently, a cohort study of 489 patients who had a vitamin D_3_ levels detected in the year before COVID-19 testing was 1.77 times greater for patients with vitamin D_3_ deficiency compared to the patients with a normal vitamin D_3_ status. These findings appear to support a role of vitamin D_3_ status for the COVID-19 risk (Meltzer et al., 2020).

The hypothesis that supplementation with vitamin D_3_ may reduce the risk of influenza and COVID-19 disease, as well the death should be examined in the trials to evaluate the correct doses, the serum 25-(OH)-vitamin D_3_ concentrations and the existence of any health concerns. There are a good model from Atlanta and Georgia in which have done the RCT on vitamin D_3_ supplementation for the ventilated ICU patients (Han et al., 2016).

There is a recommendation to take a vitamin D_3_ at 10,000 IU/day as an acceptable dose to raise circulatory concentration of vitamin D_3_ to the optimum range of 40–60 ng/ml; after 1 month this dose should be lowered to 5,000 IU/day to the sustain serum rate (Ekwaru et al., 2014; Shirvani et al., 2019). A recent study have suggested a loading doses of 200,000–300,000 IU of vitamin D_3_ to reach the optimum serum range, thereby the reducing of the risk/severity for COVID-19 (Wimalawansa, 2020).

The observation that normal vitamin D_3_ status is important for the immune system as well as for the regulation of SAR should lead to a correction of vitamin D_3_ status if a deficiency has been detected. There is no experience with the use of vitamin D_3_ in COVID-19. In addition, it should be noted that a very high doses of the upper limit of 4,000 IU (100 μg) per day of vitamin D_3_ still have the risks and may be dangerous. Since such doses might result in to the improvements in the VDR competency and could have an inhibitory impact on the immune function (Mangin et al., 2014).

## Conclusion

It is evident from the preceding discussion that vitamin D_3_ may be of benefit in COVID-19. Since the higher plasma concentrations of vitamin D_3_ is better for the protection from various viral and respiratory infections, it is reasonable to suggest that regular supplementation of vitamin D_3_ to those who are at high risk of developing various viral respiratory infections including COVID-19 need to considered seriously. To verify this proposal, double-blind placebo-controlled trials and large-scale intervention and prevention studies using vitamin D_3_ are needed. If this proposal is true it leads to the development of a simple, easily implementable method of preventing the incidence of COVID-19 and reducing its serious complications by simple oral supplementation of vitamin D_3_. Furthermore, vitamin D_3_ has several other benefits in the form of preventing rickets, improving general health, and reducing mortality due to its deficiency (though the exact cause for this association is not clear) add strength to the concept that its supplementation is warranted.

## Author Contributions

MB, VI, RM, DB interpreted the data from the literature. MB, VI, RM, JF, DB wrote the original draft. MB, VI, RM, JF, FD, LG, UD, AE-A reviewed, edited and drafted the manuscript, and approved the final version.

## Conflict of Interest

UD was employed by the company UND Life Sciences LLC.

The remaining authors declare that the research was conducted in the absence of any commercial or financial relationships that could be constructed as a potential conflict of interest.
